# Early Postmortem Changes in Pancreas: An Antemortem and Postmortem Computed Tomography Study

**DOI:** 10.7759/cureus.101444

**Published:** 2026-01-13

**Authors:** Terumi Umeda, Shohei Inui, Satori Gonoi, Wataru Gonoi, Akira Katayama, Go Shirota, Kotaro Fujimoto, Hiroyuki Abe, Keisuke Nyunoya, Mariko Kurokawa, Naomasa Okimoto, Masumi Takahashi-Mizuki, Masanori Ishida, Tetsuo Ushiku, Osamu Abe

**Affiliations:** 1 Radiology, Shimane University, Izumo, JPN; 2 Radiology, The University of Tokyo Graduate School of Medicine, Tokyo, JPN; 3 Radiology, K. International School Tokyo (KIST), Tokyo, JPN; 4 Radiology, The University of Tokyo Hospital, Tokyo, JPN; 5 Pathology, The University of Tokyo Hospital, Tokyo, JPN; 6 Radiology, Tokyo Medical University Hospital, Tokyo, JPN; 7 Pathology, The University of Tokyo Graduate School of Medicine, Tokyo, JPN

**Keywords:** computed tomography, forensic radiology, pancreas, postmortem computed tomography, postmortem imaging

## Abstract

Background: We aimed to investigate early postmortem changes of the healthy pancreas longitudinally across antemortem computed tomography (AMCT) to postmortem computed tomography (PMCT).

Materials and methods: This study included adult patients who were treated and died at a university hospital and underwent unenhanced AMCT within seven months before death, and PMCT within 24 hours after death. Included subjects had undergone pathological autopsy between 2013 and 2020 and were proven to have a healthy pancreas on autopsy. The pancreatic parenchyma on images was segmented using CT-value threshold-assisted initial contouring followed by manual slice-by-slice refinement. Their volume, density, and histogram-based distribution parameters (skewness and kurtosis) of the voxel-wise pancreatic density distribution within the segmented pancreas region of interest were measured using an image analyzer on unenhanced AMCT and PMCT. The values were compared statistically.

Results: A total of 38 participants were included in the study (male/female, 23/15; mean age, 64.1±15.1 (SD) years). The pancreatic density on AMCT (31.2 ± 8.7 HU) decreased significantly on PMCT (26.6 ± 8.1 HU). Similarly, the pancreatic volume on AMCT (63.8 ± 23.2 ml) significantly reduced on PMCT (56.8 ± 22.6 ml). Histogram skewness of the voxel-wise pancreatic density distribution significantly increased from AMCT to PMCT, whereas histogram kurtosis of this density distribution exhibited a non-significant difference.

Conclusion: Pancreatic density and volume significantly decrease in early postmortem. These findings provide a basis for interpreting normal postmortem pancreatic imaging.

## Introduction

Advanced imaging modalities such as computed tomography (CT) and magnetic resonance imaging (MRI) are increasingly used as adjuncts to traditional forensic methods in postmortem investigations [1-4]. However, radiologists often face challenges interpreting postmortem imaging due to specific and non-specific postmortem findings [5]. As international guidelines for postmortem imaging interpretation are being established, understanding the normal postmortem appearance of organs on CT is crucial [6,7].

Numerous studies have compared postmortem CT (PMCT) with antemortem CT (AMCT) to investigate early postmortem changes, mainly focusing on organ size and attenuation alterations. Several reports have documented size increases, such as brain swelling due to vasogenic edema [8] and thickening of the myocardium and major vascular walls caused by postmortem rigor mortis [9,10]. Conversely, size reductions have been observed, including decreased adrenal volume [11] and significant reductions in spleen and kidney dimensions after death [12]. Spleen volume reduction and increased attenuation were also noted in cases with normal spleens, infarcts, or tumor infiltration [13]. Changes in attenuation include postmortem increases in liver density [14] and early decreases in thyroid attenuation [15]. These findings highlight the dynamic structural and compositional changes detectable on PMCT, offering valuable insights into postmortem processes.

However, the characteristics of postmortem changes of the pancreas on postmortem imaging remain unclear. To our knowledge, no studies have described postmortem changes in the pancreas by comparing antemortem and postmortem images of the same patient. Establishing the expected direction and magnitude of normal postmortem pancreatic change is clinically and forensically relevant because diffuse hypoattenuation or mild volume change on PMCT could otherwise be misinterpreted as pathology. This exploratory study aims to longitudinally compare AMCT and PMCT in the same individuals with an autopsy-confirmed normal pancreas. We focused on quantitative changes in pancreatic volume and mean attenuation and additionally explored histogram descriptors (skewness and kurtosis) of pancreatic attenuation to characterize changes in voxel-wise density distribution.

## Materials and methods

This single-institute study was conducted at The University of Tokyo Hospital, Tokyo, Japan. The study was approved by the Institutional Review Board of the University of Tokyo Hospital (approval number: 2076), and postmortem consent to use the data for academic purposes was obtained from the bereaved families of all patients.

Study population

Adult patients who underwent AMCT within seven months before death, PMCT within 24 hours after death, and pathological autopsy between February 2013 and October 2020 were included in the study. Exclusion criteria included (i) pathological abnormalities of the pancreas, (ii) apparent abnormalities of the pancreas including main pancreatic duct dilatation on AMCT, (iii) those who showed intrapancreatic gas deposition on PMCT, (iv) those who developed pancreatic disease until death on medical records, (v) significant CT artifacts (e.g., severe beam-hardening), (vi) age under 18 years, and (vii) cases following cardiopulmonary resuscitation.

CT imaging

Antemortem CT

CT images of the pancreas were extracted from consecutive adult patients registered in the database of a university hospital. AMCT scans were obtained without contrast medium using one of the helical CT scanners (Aquilion/Aquilion ONE/Aquilion PRIME/Aquilion Precision (Canon Medical Systems Corporation, Otawara, Japan) or LightSpeed VCT/Discovery CT 750 HD/Revolution CT (GE Healthcare, Illinois, USA)) in the craniocaudal direction with the subject in the supine position. The scan parameters were as follows: slice thickness, 5 mm; slice interval, 5 mm; rotation time, 0.5 seconds; and tube voltage, 120 kVp. Automatic tube current modulation was utilized. Images were reconstructed at 0.5 mm intervals with a 350-mm field of view and a 512 × 512 image matrix. AMCT images were obtained from the patient’s last available CT examination conducted within seven months before death. Because the AMCT-to-death interval varied, sensitivity analyses restricting AMCT to scans obtained within ≤60 days before death were performed for the main paired comparisons.

Postmortem CT

All cadavers were kept in the supine position at hospital room temperature until the completion of PMCT. All postmortem CT scans were obtained without contrast medium using a 4-detector-row helical CT scanner (ROBUSTO (Hitachi Medical Corporation, Tokyo, Japan) or Aquilion (Canon Medical Systems Corporation)) in the craniocaudal direction with the cadavers in the supine position. The scanning protocol included a helical mode with a 2.5-mm slice thickness, 1.25-mm slice interval, 0.5-second rotation time, 120 kVp, and 250 mA. Images were reconstructed at a 5-mm thickness, with a 350-mm field of view and a 512 × 512 matrix.

Autopsy

All autopsies were performed by board-certified pathologists with extensive postmortem imaging experience immediately after postmortem CT. The pathologists were informed about the patient's clinical history and details regarding the circumstances of death, although they were unaware of the postmortem CT findings documented by the radiologists. The pancreas was confirmed grossly and histologically to be normal. More specifically, the pancreas was sectioned at approximately 1-cm intervals to detect any abnormalities, and a representative slice from the pancreatic tail was histologically examined with hematoxylin & eosin staining.

Image analysis

**Figure 1:**
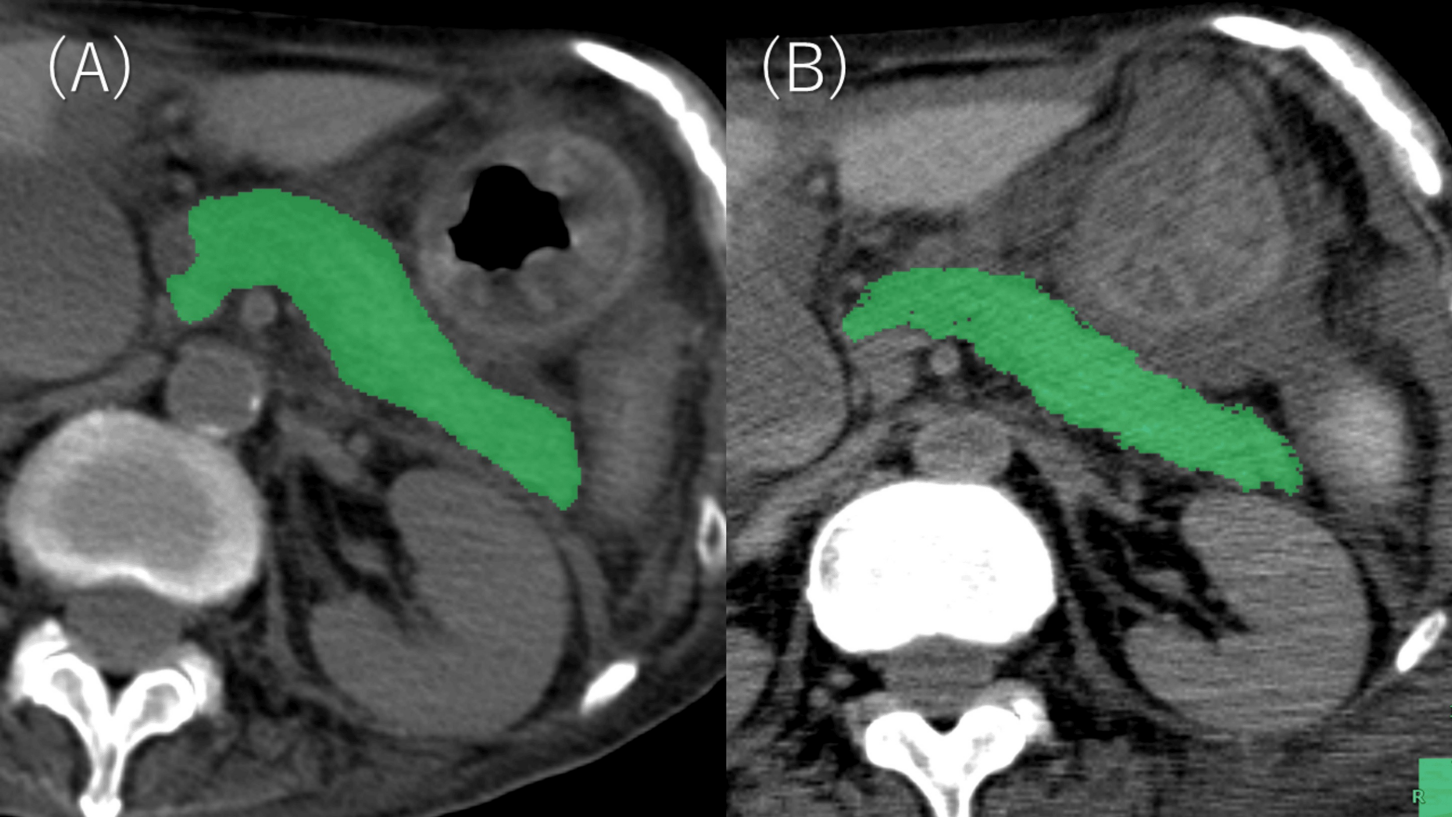
An example of regions of interest for the pancreatic parenchyma on antemortem and postmortem CT images. Regions of interest (highlighted in green) are overlaid on the whole pancreatic parenchyma in each CT slice, displayed on antemortem (A) and postmortem (B) CT images of the same patient.

All images were reviewed independently by two radiologists with eight and four years of experience (SI and AK, respectively) using SYNAPSE VINCENT (Fujifilm Corporation, Tokyo, Japan). Regions of interest (ROIs) encompassing the whole pancreatic parenchyma were first generated using vendor-provided software based on predefined CT density thresholds (Fujifilm Corporation) to obtain a rough contour. Subsequently, in every case, the ROIs were visually inspected and manually refined and corrected slice-by-slice by the two radiologists, and the final ROIs were confirmed by consensus to ensure inclusion of the pancreatic parenchyma and exclusion of adjacent structures (Figure 1). Pancreatic volume (cm^3^) and mean density (in HU) were measured using the same software. Additionally, histogram-based distribution parameters such as skewness and kurtosis describing the shape of the voxel-wise pancreatic CT density (HU) distribution within the ROIs were calculated automatically by the same software.

Skewness measures the degree of asymmetry, indicating how much a probability distribution deviates from symmetry. Kurtosis describes the tailedness of a probability distribution, reflecting whether it has heavier or lighter tails relative to a normal distribution. Skewness and kurtosis were calculated using the following formula:

$\{ x_i \}_{i=1}^{n}$ (the dataset): the collection of observed values x_1_, x_2_, …, x_n_.

$x_i$: the i-th data point in the sample.

$i$: the index of a data, running from 1 to n.

n: the sample size.

$\bar{x}$: the sample mean of the dataset.

$\displaystyle \mathrm{skewness} = \frac{\frac{1}{n} \sum_{i=1}^{n} (x_i - \bar{x})^3}{\left( \sqrt{\frac{1}{n}} \sum_{i=1}^{n} (x_i - \bar{x})^2 \right)^3}$

$\displaystyle \mathrm{kurtosis} = \frac{\frac{1}{n} \sum_{i=1}^{n} (x_i - \bar{x})^4}{\left( \sqrt{\frac{1}{n}} \sum_{i=1}^{n} (x_i - \bar{x})^2 \right)^2}$

These parameters, while not standard clinical metrics, were included to investigate quantitative changes in tissue homogeneity postmortem. 

Statistical analyses

Paired t-tests or Wilcoxon signed-rank tests, depending on whether the data followed a normal or skewed distribution, were conducted to compare each parameter between AMCT and PMCT. Stratified analysis was performed as exploratory analyses for pancreatic volume and mean density and histogram skewness and kurtosis of the voxel-wise pancreatic CT density (HU) distribution based on postmortem interval (time between death and PMCT, <12 hours or ≥12 hours), sex (male or female), and age (≤60 years or >60 years [16]). To assess robustness to variability in the AMCT-to-death interval, we performed sensitivity analyses restricting AMCT to scans obtained within ≤60 days before death for pancreatic volume and mean density. Statistical significance was defined as p < 0.05. Analyses were performed using JMP Pro 17 (JMP Statistical Discovery LLC, Cary, North Carolina, United States).

## Results

A total of 38 adults (23 men and 15 women) were included in the study. The mean age at death was 64 years (SD, 15 years). AMCT was performed within 1-208 days (median, 12 days) before death, while PMCT was performed within 2-20 hours after death (median, 8.5 hours). Sixteen individuals were 60 years or younger, while 22 were over 60. Additionally, 24 underwent PMCT within 12 hours of death, while 14 were examined after 12 hours.

Pancreatic volume

**Figure 2:**
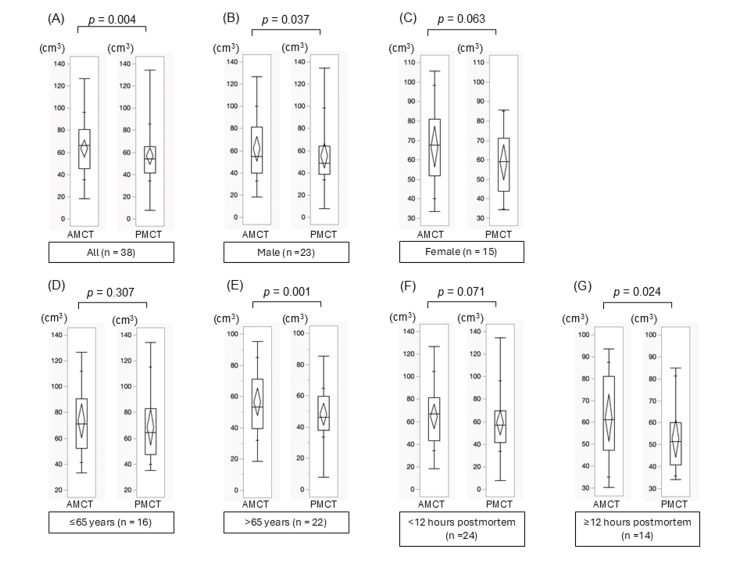
The changes in pancreatic volumes between AMCT (left column) and PMCT CT (right column) across various subgroups. (A) Full cohort (n=38), (B) Male cohort (n=23), (C) Female cohort (n=15), (D) Individuals aged ≤60 years (n=16), (E) Individuals aged >60 years (n=22), (F) Cases with CT within <12 hours postmortem (n=24), and (G) Cases with CT ≥12 hours postmortem (n=14). To compare volumes between AMCT and PMCT, paired t-tests were used. AMCT: antemortem CT; PMCT: postmortem CT

Antemortem and postmortem pancreatic volume change is illustrated in Figure 2. Total pancreatic volume decreased significantly from 63.8 ± 23.2 ml antemortem to 56.8 ± 22.6 ml postmortem (p = 0.004). An exploratory stratified analysis showed a tendency for reduction within 12 hours postmortem (p = 0.071) and a significant reduction after 12 hours (p = 0.024). In an exploratory sex-stratified analysis, male cases showed a significant reduction (p = 0.037), while female cases exhibited a non-significant tendency (p = 0.063). An exploratory age-stratified analysis revealed no significant difference in individuals 60 years or younger (p = 0.307), whereas a significant reduction was observed in those over 60 (p = 0.001).

Pancreatic density

**Figure 3:**
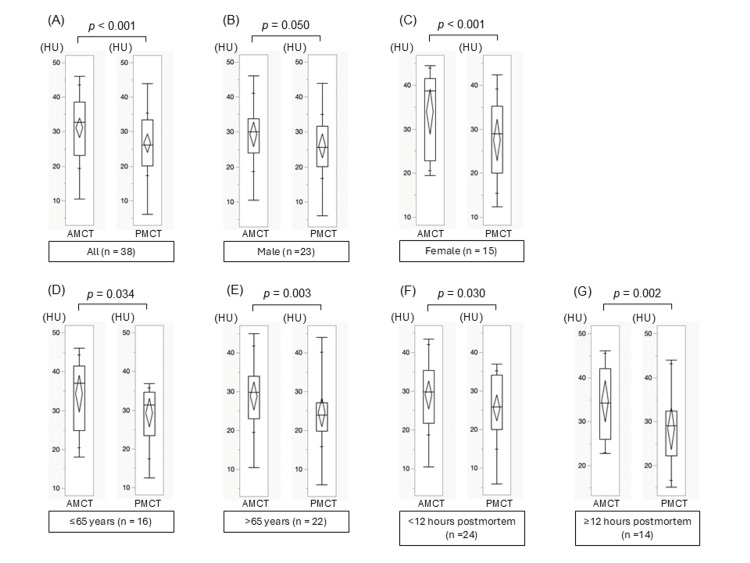
The changes in the mean pancreatic density between AMCT (left column) and PMCT (right column) across various subgroups. (A) Full cohort (n=38), (B) Male cohort (n=23), (C) Female cohort (n=15), (D) Individuals aged ≤65 years (n=16), (E) Individuals aged >65 years (n=22), (F) Cases with CT within <12 hours postmortem (n=24), and (G) Cases with CT ≥12 hours postmortem (n=14). To compare mean pancreatic densities between AMCT and PMCT, paired t-tests were used. AMCT: antemortem CT; PMCT: postmortem CT

The mean pancreatic density change between AMCT and PMCT is illustrated in Figure 3. The mean pancreatic density significantly decreased from 31.2 ± 8.7 HU antemortem to 26.6 ± 8.1 HU postmortem (p < 0.001). In exploratory stratified analyses, reductions were significant in both <12 hours (p = 0.030) and ≥12 hours (p = 0.002) postmortem intervals, as well as in both sexes (male, p = 0.050; female, p < 0.001) and age groups (≤60 years, p = 0.034; >60 years, p = 0.003).

Other parameters

In the full cohort, the skewness of the voxel-wise pancreatic density distribution increased significantly from antemortem to postmortem (p = 0.006), whereas the kurtosis of this density distribution exhibited a non-significant difference (p = 0.118). In exploratory stratified analyses, neither skewness nor kurtosis showed a significant difference.

Sensitivity analyses

Sensitivity analyses restricting AMCT to examinations performed within ≤60 days before death (n = 29) showed that pancreatic volume and mean density remained significantly decreased on PMCT compared with AMCT (p =0.032 and p = 0.011, respectively).

## Discussion

This exploratory study demonstrated significant reductions in pancreatic volume and density, as well as signficant increase in histogram skewness of the voxel-wise pancreatic density distribution within 24 hours postmortem. Exploratory time-stratified analysis indicated that these changes became more pronounced after 12 hours postmortem, suggesting a cumulative effect and potentially accelerated autolysis. Furthermore, exploratory age-stratified analysis revealed that these alterations were more prominent in individuals over 60, highlighting age-related changes in pancreatic composition and potentially increased susceptibility to autolysis.

Postmortem changes in the pancreas are characterized by morphological and histological transformations after death. These changes are crucial for understanding the postmortem interval and can provide insights into the time of death. Studies have shown that postmortem autolysis in the pancreas is influenced by various factors, including the postmortem interval and clinicopathological conditions [17]. Ultrastructural changes in the pancreas, such as cell edema, loss of glycogen granules, and nuclear chromatin condensation (apoptosis), have been observed in situ in animal models, indicating that these changes are organ-specific and occur at different rates in different tissues [18]. In particular, pancreatic acinar cells exhibit significant changes, including congestion and karyolysis, within hours after death. Histological studies have demonstrated that postmortem changes in the pancreas can be detected as early as one to two hours after death, with progressive degeneration of acinar structures and nuclear changes becoming more pronounced over time [19]. The pancreas is also subject to ischemic changes, with the exocrine parenchyma cells showing irreversible damage after a certain period of normothermic ischemia. In contrast, the islet cells of Langerhans are more resistant [20]. These findings underscore the importance of early autopsy and precise histological methods, such as epoxy resin embedding, to accurately assess postmortem changes and estimate the time of death [19]. Understanding these changes is essential for forensic investigations and can aid in distinguishing between pathological conditions and postmortem alterations [21].

The volume of the pancreas in vivo is influenced by both intrinsic factors (e.g., aging or overweight) and extrinsic factors (e.g., pancreatic diseases, surgical history, alcohol consumption) [21-24]. Diffuse pancreatic enlargement can be observed in various conditions, including inflammatory etiologies such as acute pancreatitis or autoimmune pancreatitis, as well as certain neoplasms, such as malignant lymphoma, or factors like overweight [22,25-27]. Conversely, pancreatic shrinkage may occur due to aging, chronic pancreatitis, atrophy following pancreatitis, or diabetes mellitus [16,22,28]. While no prior studies have reported postmortem changes in pancreatic volume on CT, this study revealed an average reduction of approximately 9.7% within the early postmortem period. Sex and age were not significant confounding factors in volume changes; however, volume reductions were more pronounced in individuals over 60 years old or beyond 12 hours postmortem. This is in accordance with a previous pathological study, which showed that the postmortem interval was the most crucial factor influencing postmortem autolysis [17]. Previous studies have shown that pancreatic acinar cells undergo rapid autolysis, leading to cytoplasmic vacuolization, nuclear fragmentation, and eventual disintegration of the acinar structures within hours postmortem [19]. Ischemic changes in the pancreas, particularly in the exocrine parenchyma, initially lead to cell edema, which may result in transient volume enlargement, followed by cytoplasmic condensation and shrinkage, ultimately contributing to volume reduction [20]. In contrast, as autolysis progresses, the breakdown of acinar cells and the loss of cellular integrity contribute to tissue contraction and structural collapse, which may result in volume reduction. Over time, interstitial spaces are disrupted due to enzymatic degradation, leading to further volume loss. A chronological histological study demonstrated that autolysis of the pancreatic acinar cells could be observed as early as one hour after death. In contrast, acinar cell atrophy became evident approximately 10 hours postmortem [19]. These histological observations may explain the changes observed on postmortem CT. Specifically, the combined effects of cell edema and atrophy may result in minimal overall volume reduction within the first 12 hours. However, beyond this period, the atrophic effects may become more predominant within 24 hours after death. These findings suggest that postmortem pancreatic volume changes may reflect a histopathological deterioration over time, emphasizing the importance of considering time-dependent factors when interpreting forensic imaging results.

While an increase in pancreatic density on CT is not associated with specific conditions, a reduction in density may result from various conditions, including cellular edema due to acute pancreatitis or fatty degeneration of the pancreatic parenchyma, which can occur due to aging, post-inflammatory changes, some neoplasms like pancreatic lymphoma, or diabetes mellitus [27,29]. Although no previous studies have examined postmortem changes in pancreatic density on CT, our study demonstrated an average reduction of approximately 12.3% during the early postmortem period. Sex and age were not significant confounding factors in density changes, but density reductions were more pronounced in individuals over 60 years old or beyond 12 hours postmortem. This postmortem decline in pancreatic density may be attributed to histological and biochemical changes after death. Previous studies have shown that pancreatic acinar cells undergo rapid autolysis characterized by cytoplasmic vacuolization and cell edema due to ischemic injury, which may be associated with decreased X-ray attenuation on CT within hours postmortem [18,19]. Progressive autolysis results in further disintegration of acinar structures, increasing interstitial fluid accumulation, which may further lower pancreatic density. These findings suggest that the pancreas may undergo a predictable sequence of postmortem changes and that autolysis and ischemic effects may contribute to the progressive reduction in pancreatic density. However, one should exercise caution, as ischemic changes affect the exocrine parenchyma more severely than the endocrine component, meaning the degree of density reduction may vary depending on the preservation of islet cells [20].

This study demonstrated a significant increase in pancreatic skewness from antemortem to postmortem, indicating a postmortem change in the asymmetry of the voxel-wise pancreatic density distribution within the segmented pancreas ROI. The biological mechanism behind this trend may be related to postmortem degradation and homogenization of pancreatic tissue. The pancreas consists of heterogeneous regions with varying densities due to differences in acinar cell composition, ductal structures, and fat infiltration [30]. However, after death, autolysis and ischemic changes may lead to the breakdown of cellular structures, causing a redistribution of intracellular and interstitial fluid, which may result in a more uniform density across the organ. This could explain why the skewness moved closer to zero.

This study primarily identified the pancreatic parenchyma using an automatic segmentation assistant by vendor-provided software based on CT values. In our workflow, CT-value thresholding was used only to generate an initial rough pancreas contour, and all ROIs were subsequently manually corrected slice-by-slice and finalized by consensus between two radiologists. Therefore, although attenuation-dependent thresholding combined with relatively thick slices could, in principle, bias a fully automated segmentation, systematic density-dependent exclusion of true pancreatic tissue is unlikely to explain the observed paired differences. Nevertheless, formal quantitative validation of segmentation reliability (e.g., dice similarity coefficients or interobserver intraclass correlation coefficient (ICC)) was not available in this retrospective dataset. In addition, while fixed anatomical masks are widely available in brain imaging, standardized pancreas masks are not universally available because of substantial inter-individual variability in pancreatic size and shape, making atlas-based sensitivity analyses difficult. Therefore, when interpreting the postmortem pancreatic changes observed in this study, careful consideration should be given to the potential impact of ROI selection methods on the results.

The pancreas examined in this study was pathologically normal, suggesting that the observed reduction in the volume and density and increase in skewnessrepresent expected early postmortem alterations rather than overt pancreatic disease. Because histopathology in our cohort served primarily to confirm the absence of pancreatic disease, and we did not perform quantitative time-matched histologic correlation, specific mechanistic attributions should be considered speculative. Further investigations are necessary to establish clear criteria for differentiating regular postmortem changes from pathological findings on PMCT. When interpreting PMCT, it is essential to consider pathological conditions, including medical history, and antemortem factors, such as age and time since death at PMCT acquisition.

PMCT is increasingly used in medicolegal death investigation and as an adjunct to, or triage for, autopsy. Because the pancreas can be difficult to interpret on PMCT, awareness that mild diffuse hypoattenuation and modest volume reduction may represent expected early postmortem change may help avoid misinterpreting normal postmortem change as pathology (e.g., acute pancreatitis). Conversely, focal enlargement, marked asymmetry, peripancreatic fat stranding, fluid collections, peripancreatic gas, or other supportive secondary signs should still raise suspicion for true pancreatic pathology and may warrant targeted autopsy sampling or further investigation.

Limitations are inherent in this study. First, the interval between antemortem imaging and death varied significantly, ranging from one to 208 days. To minimize potential bias, cases with pancreatic diseases in medical records and autopsy and those with apparent pancreatic abnormalities on AMCT were excluded, ensuring that the pancreatic condition remained hypothetically stable for up to seven months. As this was a retrospective study, pancreatic normality was confirmed based on routine autopsy assessment. Consequently, subtle histopathological changes may not have been identified. To address this, we performed sensitivity analyses restricting AMCT to scans obtained within ≤60 days for prime parameters before death, confirming the direction of the change was constant. Second, all cadavers were stored under standardized conditions; however, environmental biases, including temperature, may not have been avoidable. Third, the pancreas examined in this study was pathologically normal; therefore, comparisons with groups exhibiting pancreatic diseases could not be conducted. Fourth, AMCT and PMCT scans were conducted using different CT scanners, as commonly encountered in clinical and forensic settings. However, phantom calibration data were not available because this was a retrospective study using archived clinical examinations, and some scanners used during the study period have since been replaced. Thus, residual inter-scanner HU offsets cannot be fully excluded, especially given the modest mean attenuation difference. Our results should therefore be interpreted as within-subject directional changes rather than an absolute HU threshold, and future prospective studies with phantom calibration and standardized reconstruction are warranted. Fifth, quantitative analyses were performed on 5‑mm reconstructions because thin‑slice data were not consistently available in this retrospective dataset, and partial‑volume effects may have influenced pancreatic delineation and quantitative metrics. While CT‑value thresholding was used only for initial rough contouring and all ROIs were manually refined and finalized by consensus between two radiologists, residual uncertainty related to slice thickness and segmentation under postmortem hypoattenuation cannot be fully excluded. Thin‑slice reconstructions (e.g., ≤1 mm) would be preferable for pancreatic volumetry and histogram analysis, and should be adopted in future prospective studies. Regarding the distribution parameters (skewness and kurtosis), we recognize these are histogram-based statistical descriptors of the voxel-wise pancreatid density distribution, commonly used in image processing/radiomics rather than traditional medical diagnostic terms. These parameters quantitatively describe the homogeneity of tissue density distribution and can objectively assess subtle postmortem changes.

## Conclusions

Within the acute 24-hour postmortem phase, pancreatic volume and density significantly decreased. These findings are consistent with expected early postmortem pancreatic change and may help radiologists and forensic practitioners avoid misinterpreting mild diffuse hypoattenuation or modest volume reduction on PMCT as definite pathology.
